# Contribution of the Arterial System and the Heart to Blood Pressure during Normal Aging – A Simulation Study

**DOI:** 10.1371/journal.pone.0157493

**Published:** 2016-06-24

**Authors:** Elira Maksuti, Nico Westerhof, Berend E. Westerhof, Michael Broomé, Nikos Stergiopulos

**Affiliations:** 1 Department of Medical Engineering, School of Technology and Health, KTH Royal Institute of Technology, Stockholm, Sweden; 2 Department of Clinical Physiology, Karolinska Institutet, Stockholm, Sweden; 3 Departments of Physiology and Pulmonary Diseases, ICaR-VU, VU University Medical Center, Amsterdam, The Netherlands; 4 Edwards Lifesciences BMEYE, Critical Care Noninvasive, Amsterdam, The Netherlands; 5 Heart Failure Research Center, Laboratory for Clinical Cardiovascular Physiology, Academic Medical Center, Amsterdam, The Netherlands; 6 ECMO Department, Karolinska University Hospital, Stockholm, Sweden; 7 Department of Physiology and Pharmacology, Karolinska Institutet, Stockholm, Sweden; 8 Laboratory of Hemodynamics and Cardiovascular Technology, Institute of Bioengineering, Swiss Federal Institute of Technology, Ecole Polytechnique Fédérale de Lausanne, Lausanne, Switzerland; Niigata University Graduate School of Medical and Dental Sciences, JAPAN

## Abstract

During aging, systolic blood pressure continuously increases over time, whereas diastolic pressure first increases and then slightly decreases after middle age. These pressure changes are usually explained by changes of the arterial system alone (increase in arterial stiffness and vascular resistance). However, we hypothesise that the heart contributes to the age-related blood pressure progression as well. In the present study we quantified the blood pressure changes in normal aging by using a Windkessel model for the arterial system and the time-varying elastance model for the heart, and compared the simulation results with data from the Framingham Heart Study. Parameters representing arterial changes (resistance and stiffness) during aging were based on literature values, whereas parameters representing cardiac changes were computed through physiological rules (compensated hypertrophy and preservation of end-diastolic volume). When taking into account arterial changes only, the systolic and diastolic pressure did not agree well with the population data. Between 20 and 80 years, systolic pressure increased from 100 to 122 mmHg, and diastolic pressure decreased from 76 to 55 mmHg. When taking cardiac adaptations into account as well, systolic and diastolic pressure increased from 100 to 151 mmHg and decreased from 76 to 69 mmHg, respectively. Our results show that not only the arterial system, but also the heart, contributes to the changes in blood pressure during aging. The changes in arterial properties initiate a systolic pressure increase, which in turn initiates a cardiac remodelling process that further augments systolic pressure and mitigates the decrease in diastolic pressure.

## Introduction

Blood pressure changes with age [1]. During normal aging, without drug treatment, systolic blood pressure continuously increases over time, whereas diastolic pressure increases between 20 and 50 years of age, and then decreases slightly after the age of 55 [1,2]. Consequently, pulse pressure (the difference between systolic and diastolic pressure) increases over the entire period from 20 to 80 years of age [1]. The increase in pulse pressure is mainly due to stiffening of the large arteries [3], and accelerates in later years [4]. Mean pressure also increases with age [1], mainly as a result of an increased vascular resistance combined with a fairly well-preserved cardiac output [5].

The blood pressure progression with age is usually explained by changes of the arterial system alone [3]. However, changes in arterial properties that result in a systolic pressure increase induce ventricular remodelling, thus affecting cardiac structure and function [6–8]. The typical form of ventricular remodelling observed with increased afterload is concentric hypertrophy. This type of remodelling results in an increased muscle cross-sectional area that, in turn, increases pump function and has been shown to generate an additional increase in blood pressure [9]. While cardiac hypertrophy stemming from these arterial changes is widely recognized and reported, the effects of the hypertrophy on blood pressure have not been taken into account quantitatively.

In this study we hypothesise that both the arterial system and the heart contribute to the blood pressure progression during aging. According to our hypothesis, the arterial stiffness and resistance changes are the initial cause of the increased systolic blood pressure, and the cardiac hypertrophy that follows contributes to a further systolic pressure increase. The aim of this study was to capture this chain of events and to quantify the mechanisms involved in blood pressure changes during normal aging by using a Windkessel model [10] for the arterial system and the time-varying elastance model for the cardiac pump [11]. A video summary of this article is available as on online supplement (S1 Video).

## Methods

### Ventricular-Arterial Interaction Model

**Fig 1:**
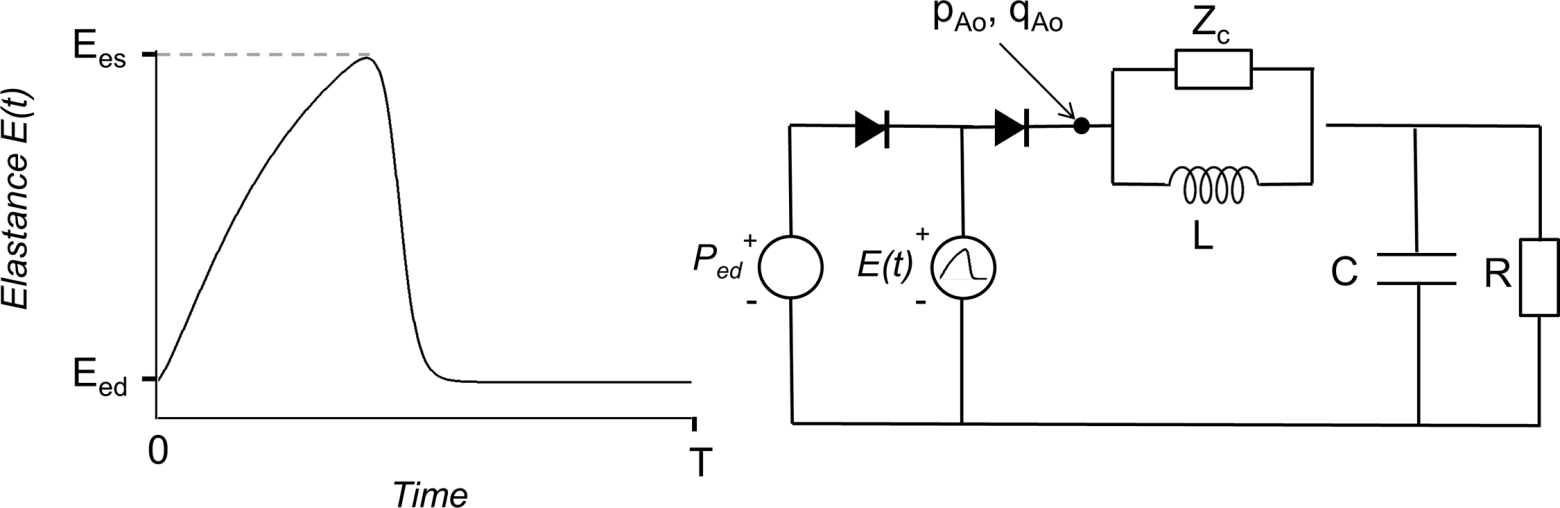
Schematic representation of the time-varying elastance model E(t) and the four-element Windkessel model. E(t) varies from its end-diastolic value (Eed) to its end-systolic value (Ees) during the heart period (T). The arterial model includes characteristic impedance (Zc), inertance (L), total arterial compliance (C) and vascular resistance (R). Voltage and current at the beginning of the Windkessel model represent aortic pressure (pAo) and flow (qAo), respectively.

The contributions of the arterial system and the heart to aortic blood pressure were quantified using a ventricular-arterial interaction model, previously described and validated [9,12]. A scheme of the model is shown in Fig 1. The systemic arterial tree is represented by the four-element Windkessel model [10], which has as its model parameters total arterial compliance (C) representing the inverse of arterial stiffness, vascular resistance (R), aortic characteristic impedance (Z_c_) and total inertance (L).

The left ventricle (LV) is represented by the time-varying Elastance model, E(t) [11–14]. During each cardiac cycle, E(t) increases from its diastolic value to its systolic value and then returns to its diastolic value again. E(t) varies over the cardiac cycle according to a periodic function that, after normalization, is similar under different conditions, such as hypertrophy [14]. This E(t) can be approximated by the periodic “double-Hill” mathematical function as described by Stergiopulos et al. [12]. The cardiac model parameters are end-systolic LV pressure-volume relationship (Ees) representing myocardial contractility, end-diastolic LV pressure-volume relationship (Eed) representing diastolic myocardial stiffness and end-diastolic LV pressure (Ped). Increases in muscle cross-section and thus wall thickness cause an increase in Ees and Eed. Cardiac valves include a small resistance (0.003 mmHg∙s/mL), representing the resistance to flow through the open valve leaflets, and a small inertance (3∙10^−5^ mmHg∙s2/mL), representing blood inertia. Valves can be either fully open or fully closed and allow only forward flow.

Ascending aortic pressure and flow are computed at the connection between the aortic valve and the four-element Windkessel model. The following output variables are calculated: LV pressure, LV volume, ascending aorta blood pressure and aortic flow.

### Initial Model’s Parameters

The model parameters for a normal 20 years old adult were chosen in order to reproduce physiological pressure and flow curves. The arterial model parameters at this age were C = 2.8 mL/mmHg, R = 0.8 mmHg∙s/mL, Z_c_ = 0.02 mmHg∙s/mL and L = 0.005 mmHg∙s^2^/mL [9,15]. C and R were varied with age during simulations (for each decade between 20 and 80 years, see below), Z_c_ was varied in proportion to 1/$\sqrt{C}$ [9] and L was kept constant for all ages.

Cardiac parameters were also chosen as normal values at 20 years of age, with Ees = 1 mmHg/mL and Eed = 0.025 mmHg/mL, and end-diastolic ventricular pressure was taken as 5 mmHg. Heart rate was set at 67 beats/min (R-R-interval 0.89 s) and assumed to be independent of age [2]. The parameters of the E(t), related to rising time during systolic contraction and relaxation time during diastole, were chosen according to previous studies [12,13] and assumed unchanged for all ages.

### Arterial and Cardiac Parameters Changes with Age

Arterial stiffness (inverse of compliance) increases with age mainly due to structural changes of the arterial wall [3]. This increase in stiffness was estimated from pulse wave velocity, which increases by approximately a factor of two over this age range [2,16], corresponding to a decrease in compliance by a factor of four [17] (linear decrease from compliance at 20 years to 0.7 mL/mmHg at 80 years). In order to generate a physiological central mean pressure at a young age [18], Resistance increase was chosen 5% per decade based on the work by Segers et al. [19], thus increasing linearly from 0.8 mmHg s/ml to 1.04 mmHg s/ml.

Calculations were performed for each age group. First, only changes in total arterial compliance and vascular resistance were taken into account. Then the cardiac parameters and the diastolic filling pressure (Ees, Eed and Ped) were modified according to two rules. The *first rule* was to normalize ventricular wall stress [20,21]. The Law of Laplace [6,7,22] relates wall stress σ to LV pressure P_lv_, LV radius r_i_ and wall thickness h according to the formula

$$\sigma =\frac{P_{LV}∙r_{i}}{2h}.$$ (1)

**Fig 2:**
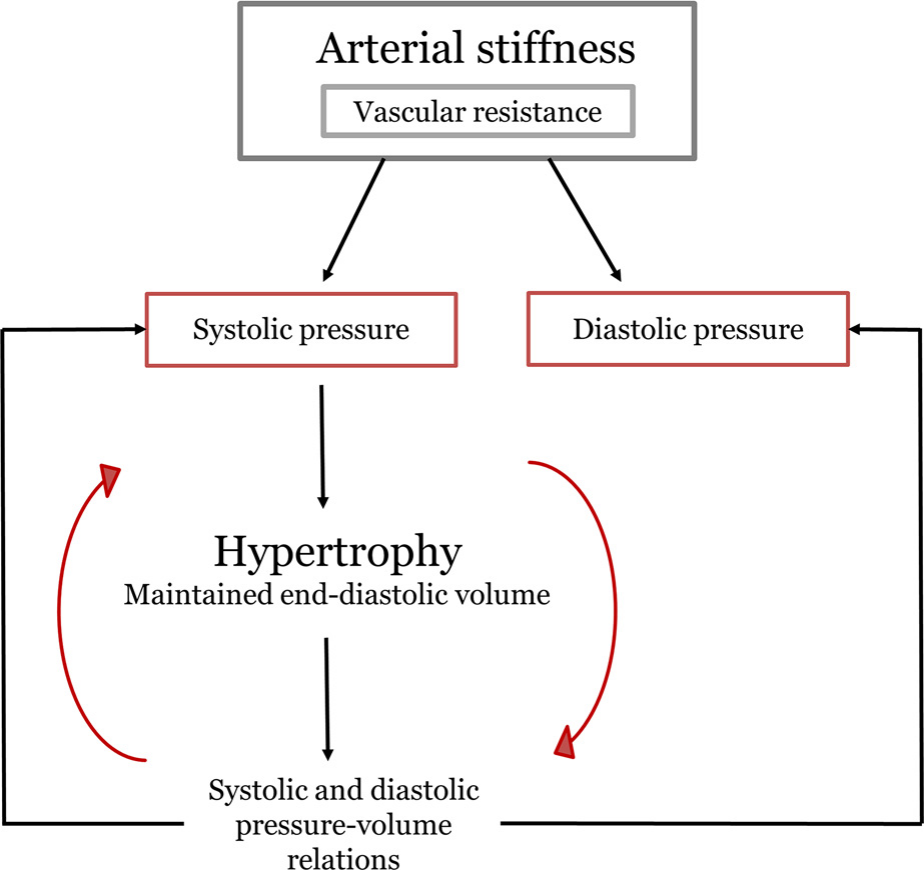
Scheme of the events occurring during normal aging, used to guide the model’s parameters selection. The loop Hypertrophy–Pressure-volume relations–Systolic pressure is repeatedly carried out until stable pressures and volumes are obtained.

In order to preserve wall stress in Eq (1) and considering a constant lumen radius as in concentric remodelling [7], wall thickness increase must be proportional to the increase in systolic pressure. Successively, we assumed that the increased wall thickness causes a directly proportional increase of both Ees and Eed (ΔPsys = ΔEes = ΔEed). The *second rule* was that Ped was increased such that the end-diastolic volume remained constant, as reported by Lakatta [5]. The increased wall thickness resulted in an increase in systolic pressure that, in turn, caused more hypertrophy. Ees, Eed and Ped were then increased again, and so on until equilibrium was obtained (changes less than 1 mmHg or 1 mL). A summary of the reasoning that guided the parameter selection is presented in Fig 2. Simulation results for aortic blood pressure over the age range were then compared with clinical pressure data from the Framingham Heart Study reported by Franklin et al. [1], using the mean value between normotensive groups 1 and 2 (Figure 3 in Franklin et al. [1]). In order to account for systolic pressure amplification at the brachial level, we added (for each decade) the average systolic pressure amplification reported by Avolio et al. [23] to the calculated central aortic systolic pressure.

To quantify the difference between the model and the population data, a normalized root-mean-squared error (RMSE) was calculated considering the relative difference between the model and the reference values, for systolic and diastolic pressure.

## Results

The schematic of the approach is given in Fig 2. The parameters that changed with age during the simulations, either prescribed (arterial parameters based on data from literature) or computed (cardiac parameters derived from the two physiological rules), are reported in Table 1.

**Table 1:** Arterial and cardiac model parameters at different ages.

	Arterial parameters (prescribed)			Cardiac parameters (computed)		
Age (years)	C (mL/mmHg)	R (mmHg∙s/mL)	Zc (mmHg∙s/mL)	Ees (mmHg/mL)	Eed (mmHg/mL)	Ped**(mmHg)**
20	2.80	0.80	0.020	1.00	0.02500	5.00
30	2.45	0.84	0.021	1.03	0.02575	5.10
40	2.10	0.88	0.023	1.09	0.02725	5.40
50	1.75	0.92	0.025	1.16	0.02750	5.75
60	1.40	0.96	0.028	1.24	0.03100	6.10
70	1.05	1.00	0.033	1.35	0.03375	6.65
80	0.70	1.04	0.040	1.51	0.03775	7.40

Abbreviations: arterial compliance (C), vascular resistance (R), characteristic impedance (Zc), end-systolic elastance (Ees), end-diastolic elastance (Eed), end-diastolic pressure (Ped).

**Fig 3:**
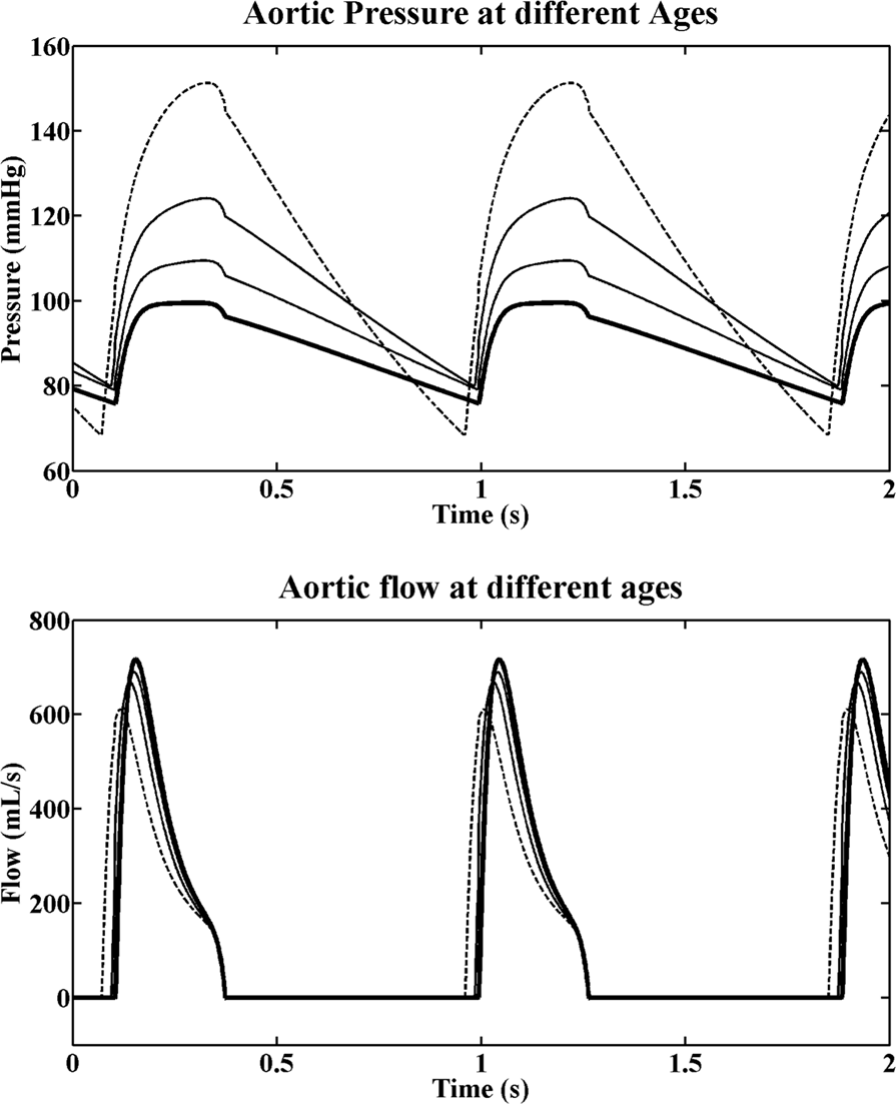
Aortic pressure and flow profiles. The curves were computed at 20 (bold), 40, 60 and 80 (dashed) years of ages, when considering both the contribution of the arterial and cardiac changes.

Fig 3 shows simulated pressure and flow wave shapes at 20, 40, 60 and 80 years of age. Aortic pressures rises more steeply at older ages due to higher arterial stiffness and higher characteristic impedance. The systolic pressure is reached later in the ejection phase when age increases. Simulated flow profiles are similar for all ages, with a slight decrease in peak flow and a 12% increase in ejection time between 20 and 80 years. A 5% age-dependent increase in left ventricular ejection time was reported in elderly subjects, as compared with young individuals [24].

**Fig 4:**
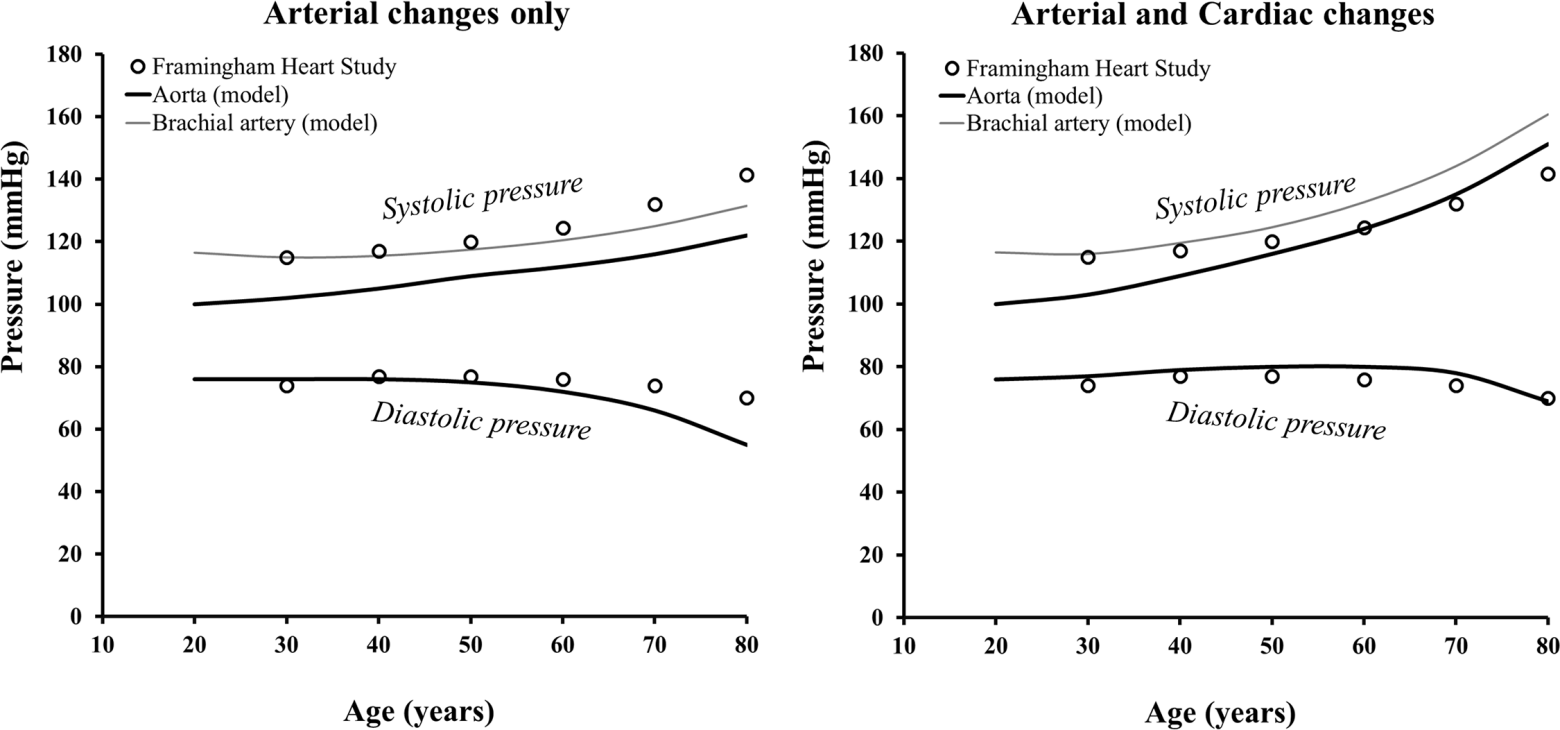
Comparison between the model’s aortic systolic and diastolic pressures changes with age and population data for brachial pressure. (On the left) Simulations for arterial parameter changes only and (on the right) for arterial and cardiac parameter changes combined. Parameters for the arterial changes are prescribed, whereas parameters for the cardiac changes are computed through physiological rules. The thin grey line represents derived brachial systolic pressure, obtained accounting for the amplification between aortic and brachial systolic pressure based on data reported by Avolio et al. [23].

A comparison between the model’s systolic and diastolic aortic pressure as a function of age and population data is presented in Fig 4. Results are presented for arterial changes only and for arterial plus cardiac changes combined. When taking into account arterial changes only, systolic blood pressure increased from 100 to 122 mmHg and diastolic blood pressure decreased from 76 to 55 mmHg. These calculated blood pressure values do not conform to the arterial pressure values reported in the Framingham Heart Study [1], as can be seen in Fig 4. In this case, when only arterial changes were applied, the calculated stroke volume decreased by approximately 20% between the ages of 20 and 80 years.

When also taking cardiac changes into account, systolic aortic pressure varied from 100 to 151 mmHg and diastolic aortic pressure from 76 to 69 mmHg in the evaluated age range. These values are close to the ones reported in the Framingham Heart Study for normotensive subjects (Fig 3). The RMSE for the simulations with arterial and cardiac changes combined was lower than for arterial only (5.1 vs 10.7%). The contribution of the ventricular-arterial interaction can also be seen in the greater widening of pulse pressure with age, similar to the population data. If considering an additional systolic pressure increase to account for brachial artery pressure amplification (gray line in Fig 4), the model’s systolic pressure better mimics the population data at young ages. At older ages, when accounting for amplification, the model underestimates systolic pressure changes when considering only arterial changes and overestimates systolic blood pressure when considering arterial and cardiac changes. The RMSE between the model for brachial pressure and the population data was 7.7% for arterial changes only and 5.9% for arterial and cardiac changes combined.

As can be seen in Table 1, Ees and Eed increased by 51% between 20 to 80 years old in order to normalize LV stress, and contributed to the increase in systolic aortic pressure. Ped also increased, in agreement with previously reported data [25].

## Discussion

This study shows in a quantitative manner that not only the arterial system, but also the heart, substantially contributes to the blood pressure changes in normal aging (Figs 3 and 4 and Table 1). The changes in arterial properties (i.e. arterial stiffness and vascular resistance) are assumed to initiate the blood pressure changes. Subsequently, a cardiac remodelling process further contributes to the increase in systolic arterial pressure. Compensated hypertrophy and preservation of LV volumes (by increased filling pressure) may be regulatory mechanisms involved in this remodelling process in order to prevent cardiac output from decreasing. Arterial changes alone did not correctly reproduce the diastolic pressure progression with age, which considerably decreased after the age of 60 years. By including cardiac adaptation, diastolic pressure could be better preserved till the age of 80 years. Thus the role of the heart was particularly important in bringing diastolic blood pressure changes closer to the reference values (Fig 4).

The cardiac and arterial contribution to mainly systolic hypertension in middle-aged individuals has previously been quantified [9]. However, the present study, to the best of our knowledge, is the first to: (I) describe the time evolution of blood pressure changes in normal aging over a wide age range, (II) quantify the contributions of both the heart and the arterial system to pressure, and (III) compare the results with a large population study such as the Framingham Heart Study [1].

Recent efforts have been made to establish reference values for blood pressure with age from multiple-centre studies [26], which partially differ from the data reported by Franklin et al. [1]. We chose to use the values from the Framingham Heart Study as reference values for our simulations since this latter is a longitudinal study, rather than a cross-sectional study, on subjects not treated with antihypertensive medication.

The simplified approach we used for both the arterial load and cardiac adaptation captured the main features of pressure changes with age fairly well (Fig 4). Since the output of the model was aortic pressure, we included an amplification value at each decade [23] to improve comparison of the model-derived pressures with clinical brachial measurements. Some of the differences between the model derived pressure and clinical data at older ages can be explained by other ventricular remodelling processes (such as fibrosis) that were not taken into account, as further discussed in the “Cardiac Changes” section.

### Arterial changes

Arterial stiffness has been extensively studied since its increase is an independent risk factor for cardiovascular disease [27]. Pulse wave velocity is a well-established method for estimating central arterial stiffness and it is related to the total arterial compliance [17]. The changes in arterial stiffness with age (simulated by changing the total arterial compliance parameter of the Windkessel model) were based on recent studies on large population samples, where a pulse wave velocity increase by a factor of two over the investigated age range was reported [2,16,28], corresponding to a decrease by a factor of four in total arterial compliance between 20 and 80 years [17]. The applied linear decrease in compliance corresponds to a stiffness increase accelerating with age, as previously reported [4]. Changes in characteristic impedance did not have a significant effect on systolic and diastolic pressure (less than 2 mmHg), but play a role in the blood pressure waveform by generating a more sharp or smooth pressure profile during the time period when the aortic valve is open. The effect was similar to what seen in previous studies [29].

The increase in vascular resistance with age is less well documented than changes in stiffness. The vascular resistance at 20 years of age was chosen to generate a normal mean pressure for a young individual [1]. From this initial value, we used data by Segers et al. [19], where an increase of 5% per decade was reported. This increase in vascular resistance helps to maintain coronary perfusion, compensating for the diastolic pressure drop caused by the increase in arterial stiffness.

Large arteries not only change their mechanical properties with age, but also their shape and geometry [3,30]. The complex arterial remodelling may result in inertance changes, which were not taken into account in this study since the pressure losses due to shape are small and the contribution of inertance to systolic and diastolic blood pressure was found to be small compared with the contribution of C and R [9,12].

### Cardiac changes

Information about cardiac systolic and diastolic function and filling pressure changes with age is limited, especially in the normal population, since invasive measurements are usually needed to obtain quantitative information. To overcome this problem, we considered physiological rules supported by population data to account for cardiac changes with age. Systolic pressure is considered one of the main determinants of concentric hypertrophy [6,7,22] and in order to normalise ventricular wall stress, we assumed an increase in Ees proportional to systolic pressure. In addition to systolic pressure, hypertrophy may also be related to ventricular wall stress [31] and diastolic strain [32]. However, a full analysis would require the use of a ventricular model computing wall stress and the introduction of more assumptions and parameters.

Hypertrophy influences diastolic properties as well, since a thicker myocardium results in a stiffer ventricle, which is more difficult to fill. We applied an increase in Eed proportional to the increase in Ees at different ages, i.e. both elastance values were considered proportional to the wall thickness. More complex remodelling processes (such as fibrosis) are also involved in the age-related LV stiffness increase as a result of molecular, cellular and extracellular matrix changes [33,34]. These changes could potentially result in an even larger increase in Eed. A significant component of age-related LV stiffening was shown to be independent of the increased arterial load and therefore likely related to myocardial mechanical changes [35]. The mechanisms causing the age-related LV stiffening remain unclear. Data on ventricular dimensions and diastolic filling pressure during normal aging could help in more accurate modelling.

The assumption of an unchanged end-diastolic volume during cardiac adaptation (rule 2) is not only supported by the close agreement with systolic and, especially, diastolic pressure in normotensive subjects [1], but also by an age dependent increase in filling pressures (Table 1) similar to population data reported by Redfield et al. [25] (data are reported for a mixed population, not only for healthy individuals). Also the computed increase in Ees and Eed with age is in agreement with data reported by Redfield et al. [25]. However, caution should be used when doing a quantitative comparison between our simulation results and these population data, since these latter are based on non-invasive and fairly approximate methods.

Heart rate was kept constant for all the decades during simulation since multiple studies have reported no changes of resting and average heart rates with age [2,36] (data in Scuteri et al. [2] are reported in Fig S9 in their supplement).

### Limitations of the Model

In this study, the contribution of the arterial system and the heart to blood pressure changes was based on a mathematical model, which is a simplification of reality.

A recent study using cardiac magnetic resonance in healthy individuals [37] has shown a decrease of 10% in cardiac index with age (21–81 years), which would correspond to a 10% decrease in SV volume, when heart rate and body surface area are kept constant. The decrease in cardiac index could be explained by a decrease in metabolism, as suggested by Carlsson et al. [37] or by development of fibrosis [34], as previously mentioned. To obtain such a decrease in cardiac index in the simulations, while generating the same increase in blood pressure as in Fig 3, would require an even bigger increase in C and R. A similar magnetic resonance study [38] has reported no significant changes in wall stress during aging, in a healthy population. The observed preservation of wall stress as well as the close agreement between simulated pressure changes and clinical measurements support the choice of the physiological rules applied, despite their simplicity.

We have assumed a constant shape of the elastance function E(t) (i.e. constant rising and falling time [14]), although progressive fibrosis and hypertrophy during aging may result in slight widening of the QRS-complex, slowing cardiac conduction and therefore influencing the timing of both contraction and relaxation. The magnitude of these changes, however, is small in normal aging. In Fig 3, the aortic valve opens earlier at 80 years compared with at 20 years because the E(t) time-pattern is similar while the diastolic pressure is lower at 80 years of age.

The Windkessel model mimics the arterial load well but does not take wave transmission and reflections into account. Arterial reflections do influence the pressure amplitude and particularly the shape of pressure and flow waveforms. The use of a distributed model of the arterial tree could help investigating the effect of wave transmission during aging, but would require detailed information about the arterial system geometry and arterial stiffness at all locations for different ages.

The dicrotic notch is not correctly reproduced by the model since valve closure is instantaneous. This limitation of the model only minimally influences our results and conclusions since we compare peak aortic pressure (systolic pressure) and minimum aortic pressure (diastolic pressure) with the population data.

### Generalization

Quantification of arterial blood pressure during conditions other than normal aging is feasible but requires information about the arterial and cardiac parameters in those specific cases. The aim of this study was limited to blood pressure changes during normal aging. In systolic hypertension, both cardiac and arterial changes will be more pronounced than in the present study. In decompensated left heart failure, the left ventricle will dilate in response to the age-related increase in afterload. These pathological states are beyond the scope of this study.

Arterial and cardiac properties also depend on gender [2,19,25,39]. This could explain gender-related differences in blood pressure, heart failure incidence and life expectancy between men and women, and is therefore of high clinical relevance. However, clear trends and statistically significant differences are not yet well documented over the wide age range of arterial and arterial properties considered in this study. The input data we used are derived from population studies in which both genders were included and should therefore be considered as gender independent.

Endurance sports and heavy exercise in athletes may be associated with LV hypertrophy [40]. In the athlete’s cardiovascular system, hypertrophy goes along with low heart rate, normal resting blood pressure and higher arterial compliance [41]. We considered these conditions to be beyond the scope of this study.

## Conclusions

This study shows that both the arterial system and the heart contribute to the blood pressure changes in normal aging and quantifies the arterial and cardiac contributions to blood pressure over a wide age range (20 to 80 years).

The changes in arterial properties initiate a systolic pressure increase, which in turn initiates a cardiac remodelling process that further augments the systolic pressure and mitigates the decrease in diastolic pressure.

The close agreement between simulated pressure changes and published data supports the idea that the approach presented may be applicable to the analysis of arterial and cardiac changes in both health and disease. More data on arterial and cardiac function in aging would help future refinement of the model and application to other conditions.

## Supporting Information

S1 Video Video abstract. The research questions and the main results of the study are explained in this three-minute video summary.
(MP4)

## Abbreviations

C: total arterial compliance
R: vascular resistance
Zc: aortic characteristic impedance
L: total inertance
LV: left ventricle
E(t): time-varying elastance
Ees: end-systolic pressure-volume relationship
Eed: end-diastolic pressure-volume relationship
Ped: end-diastolic pressure
